# Supplementary material for: Protein synthesis rate is the predominant regulator of protein expression during differentiation
Source: Mol Syst Biol. 2013 Sep 17;9:689. doi: 10.1038/msb.2013.47 (PMC3792347; doi:10.1038/msb.2013.47)
Supplement: Supplementary Materials [file msb201347-s1.docx]

Supplementary Materials

# Supplementary Tables

| **Supplementary tables** | **Title** |
| --- | --- |
| Supplementary table 1 | Protein expression changes during THP-1 differentiation |
| Supplementary table 2 | Protein expression changes during C2C12 differentiation |
| Supplementary table 3 | Synthesis and degradation rates in differentiating or proliferating cells |
| Supplementary table 4 | Enriched biological processes and proteins of macromolecular complexes with similar synthesis or degradation rates |
| Supplementary table 5 | Synthesis and degradation rates of macromolecular sub-complexes |
| Supplementary table 6 | Partial least square regression |
| Supplementary table 7 | 2D enrichment of biological processes between the synthesis rates of differentiating and proliferating cells |
| Supplementary table 8 | 2D enrichment of biological processes between the degradation rates of differentiating and proliferating cells |
| Supplementary table 9 | Enrichment of biological processes for proteins that display change in synthesis or degradation rate |

# Supplementary Figures

Supplementary Figure 1: 2D enrichment analysis of the protein expression in differentiating C2C12 and THP-1 cells

2D enrichment analysis was performed similarly to Cox et al.(Cox & Mann, 2012) (P<0.05) and detects proteins in the two cell lines that display consistent behavior in any of the data dimensions versus the rest of the proteins in the dataset. The cellular processes located on the diagonal suggest these are similarly regulated in THP-1 and C2C12 cells, whereas the processes located off-diagonal are regulated differently between THP-1 and C2C12 cells.

Supplementary Figure 2: Spread of relative synthesis and degradation rates of all the proteins detected in the individual fractions eluting from the SEC column.

Plotted are the median and standard deviation of the M/L ratio (A) and the H/L ratio (B) of the individual proteins in the individual fractions.

Supplementary Figure 3: Psma and Pac1-4 co-elute in size exclusion chromatography.

The alpha subunits of the proteasome core particle typically elute from size exclusion chromatography in at least three distinct peaks, with the peak around fraction 29 co-eluting with the proteasome assembling chaperones (PAC1-PAC4).

**Supplementary Figure 4 Predicted versus observed protein expression changes using partial least square (PLS) regression**

Using the mean of the three biological replicates for protein synthesis rate, protein degradation rate and mRNA expression change, a PLS regression model was developed that could account for 74% of the variation in the experimentally confirmed protein expression rates.

**Supplementary Figure 5: Partial least square (PLS) regression of the protein expression change in C2C12 cells**

PLS regression using the synthesis and degradation rates of the proteins to predict protein expression in differentiating C2C12 cells. For the boxplots, the red line denotes the median, the box the 1^st^ and 3^rd^ quartile and the whiskers span the most extreme values. B) The significance of the different parameters in the PLS regression using the Wilcoxon-Mann-Whitney test between the R^2^-values of the three biological replicates of the synthesis and degradation parameters. Similar to the THP-1 cells, the synthesis rate is a much better predictor than the degradation rate for changes in protein expression.

**Supplementary Figure 6 Post transcriptional regulation serves as a fine-tuning mechanism of protein expression**

Predicted protein expression using RNA expression only (black circles) or RNA expression and synthesis rate (red triangles). The inclusion of the synthesis rates leads to better prediction of the observed protein expression through compensating for post transcriptional regulation such as miRNAs. Notice these changes are minor, confirming earlier observations that miRNA are making fine-scale adjustments to the protein expression (Baek *et al*, 2008; Selbach *et al*, 2008).

Supplementary Figure 7: Regulation of protein synthesis rates in response to differentiation in C2C12 cells

2D enrichment analysis (P<0.05) of the relative synthesis rates in proliferating and differentiating C2C12 cells. Most of the cellular processes are located off the diagonal suggesting that the relative synthesis rate is regulated in response to differentiation.

Supplementary Figure 8: Regulation of protein degradation rates in response to differentiation in C2C12 cells

2D enrichment analysis (P<0.05) of the relative degradation rate in proliferating and differentiating C2C12 cells. Compared to the spread in Supplementary Figure 7, most of the cellular processes are clustered more tightly to the diagonal, suggesting that the relative degradation rate is regulated fairly little in response to differentiation.

Supplementary Figure 9: The regulation of the synthesis and degradation rate of the proteins involved in cell cycle in response to differentiation

Scatterplots of A) degradation and B) synthesis rates for cell cycle-related proteins in proliferating vs. differentiating THP-1 cells. Degradations rates have a higher correlation coefficient suggesting that there is limited regulation of the degradation rates but dramatic regulation of the synthesis rates of individual proteins in response to differentiation.

# Supplementary methods

## Sample preparations

### Proteome expression changes during differentiation of THP-1 and C2C12 cells

THP-1 cells were grown in RPMI media with 10 % dialyzed fetal bovine serum (FBS), 1 % glutamine, 1 % non essential amino acids, 1% penicillin/streptomycin and either (L-[U-^13^C_6_,^14^N_4_]arginine and L-[^2^H_4_]lysine or L-[U-^12^C_6_,^14^N_4_]arginine [^1^H_4_]lysine or L-[U-^13^C_6_,^15^N_4_]arginine and L-[U-^13^C_6_,^15^N_2_]lysine (Cambridge Isotope Labs, Cambridge, MA). Cells were grown for at least five doublings to ensure 100% incorporation of labeled amino acids, before differentiated by applying 25 nM PMA for the given amount of time. Adherent and suspended cells were harvested by scraping and centrifugation at 600 relative centrifugal force (rcf) before being washed three times in PBS, lysed in 1 % deoxycholate, boiled for 5 min and, finally, equal amounts of protein from the three cell populations were mixed together.

C2C12 cells were grown in Dulbecco’s Modified Eagle’s medium (DMEM) with 20 % dialyzed FBS, 1 % glutamine, 1% penicillin/streptomycin and either (L-[U-^13^C_6_,^14^N_4_]arginine and L-[^2^H_4_]lysine or L-[U-^12^C_6_,^14^N_4_]arginine [^1^H_4_]lysine or L-[U-^13^C_6_,^15^N_4_]arginine and L-[U-^13^C_6_,^15^N_2_]lysine (Cambridge Isotope Labs, Cambridge, MA). The cells were split from 50% confluence to 100 % confluence at time 0 h while being transferred to similar media as described above except that 2 % FBS was added. Afterwards, the cells were washed three times in PBS, scraped off the plate in PBS, lysed in 1 % deoxycholate, boiled for 5 min and, finally, equal amounts of protein from the three cell populations were mixed together.

### Measurement of relative synthesis and degradation rates

Two populations of cells were grown in the light and the medium media as described above for the respective cell lines. The “light” cells were harvested at time 0 h and frozen, while simultaneously the “medium” cells were washed three times with PBS and transferred to the “heavy” form of amino acids. After 48 h of proliferation or differentiation, (24 h for the proliferating C2C12 cells, which grow much faster) the cells were harvested, lysed and mixed with the “light” lysate before proceeding as below.

### Lysate preparation

The lysates were treated with benzoase before being reduced, alkylated and digested as previously described (Rogers & Foster, 2007) and then a total of 100 μg was separated by isoelectric focusing (Agilent technology) according to the manufactures instructions. The peptides were subsequently cleaned up(Rappsilber *et al*, 2007) and analyzed by LC-MS/MS, as previously described (Kristensen *et al*, 2012).

### Identifying the synthesis and degradation rates of proteins in macromolecular sub-complexes

Two populations of C2C12 cells were fully incorporated with “light” and “medium” amino acids before the “medium” population was switched to media containing “heavy” amino acids for 24 h. The “light” and “medium/heavy” cells were subsequently mixed together in a 1:4 ratio (to correct for the growth of the light cells), before the cells were lysed with a Dounce homogenizer in SEC buffer (20mM Tris, 50mM sodium acetate, 50 mM KCl) including Halt protease and phosphatase inhibitors cocktail (Thermo Scientific). The lysate was concentrated using ultrafiltration (100,000 molecular weight cutoff, Sartorius Stedim) and loaded onto on the preparative HPLC (Agilent technology) equipped with HPLC-SEC columns (300x7.8 mm Yarra-4000 (Phenomenex)) and fractionated into 48 fractions at 8°C, at a flow rate of 0.5 mL/min. The proteins of the individual fractions were digested to peptides and analyzed by mass spectrometry as described previously (Kristensen *et al*, 2012).

## Data analysis

### Data processing of the MS data

Tandem mass spectra were extracted, searched and quantified by MaxQuant (v2.3.0.5)(Cox & Mann, 2008). The search was performed against Uniprot (proteomes) human (69906 sequences) 21/6/2011 or mouse (55269 sequences) 20/4/2012 with common serum contaminants and enzyme sequences added, 1% FPR on protein and peptide levels, trypsin/P cleavage rule with a maximum of 2 missed cleavages, 0.5 Da tolerance for MS/MS, Carbamidomethylation on cysteines as the sole fixed modification and oxidation of methionines and acetylation on protein N-terminal as variable modifications. Finally, we used a ratio count of 2 for quantification and the match-between-run-feature with a window of 2 min for quantification.

### Identifying enriched signatures for differentially expressed proteins

Proteins whose abundance changed significantly during differentiation were identified by applying ANOVA between the five time points with the following settings (Permutation-based FDR, P=0.05, S0=1, 250 randomizations) using Perseus. Increasing and decreasing proteins were defined by clustering the data into two clusters using fuzzy C mean clustering by the following settings (c=2, m=2). Functional enrichment analysis was done using the Fisher exact test (P=0.05) the Benjamini-Hochberg correction with a minimum of 5 proteins per category using GPROX(Rigbolt *et al*, 2011).

To investigate if proteins that were changing in expression were different in abundance from the non-regulated proteins, a Wilcoxon-Mann-Whitney test was applied to compare summed up eXtracted ion Current of the two groups at 0 h differentiation.

2D enrichment analysis (P<0.05) of the biological processes (Uniprot Keywords) that were enriched in any dimension of protein expression changes after 48 h in THP-1 and C2C12 cells were generated using Perseus according to (Cox & Mann, 2012), with a Benjamini-Hochberg FDR for truncation.

### Data processing of synthesis and degradation data

The medium/light and heavy/light ratios were normalized by dividing by their median values before they were log_2_ (-log_2_ for medium/light) and Z-transformed. This approach to identifying relative synthesis and degradation rates resembles that used in cDNA microarray processing where a Z-transformation is also used to compare transcript changes between different experiments(Cheadle *et al*, 2010).

To identify proteins whose relative synthesis or degradation rates were significantly altered, we performed two-sided T-tests (P<0.05, S0=1) between the normalized data in proliferating and differentiating cells, where a Permutation-based FDR (250 randomizations) was used for truncation.

We investigated the characteristics for proteins with similar synthesis and degradation rates by measuring the Euclidian distances in synthesis/degradation space between all the proteins assigned to the same biological process (using Uniprot keywords). The P-value was calculated using Wilcoxon-Mann-Whitney test, to test if the distances in a biological process were significantly different from distances between random chosen proteins (50 iterations). A similar approach was used to investigate if there were any proteins in a macromolecular complex (defined by the CORUM database(Ruepp *et al*, 2010)) that displayed significantly different synthesis/degradation rates than the rest of the proteins in a macromolecular complex. Here, Euclidian distances were calculated among all synthesis/degradation rates of the proteins in a complex and Wilcoxon-Mann-Whitney test was applied to investigate if any proteins displayed significantly different distances than the rest of the proteins.

To investigate if proteins in macromolecular complexes (defined by the CORUM database(Ruepp *et al*, 2010)) display more similar synthesis and degradation rates than the rest of the proteins, we calculated the Euclidian distances between all proteins in a macromolecular complex and investigated if these distances to proteins not participating in macromolecular complexes were significantly smaller by Wilcoxon-Mann-Whitney test.

### Identifying enriched signatures for proteins with fast/slow degradation rates

Degradation rates measured in proliferating human THP-1 and mouse C2C12 cells were paired between the two organisms based on their unique gene names taken from the HGNC homepage (http://www.genenames.org/)(Gray *et al*, 2012). The proteins were considered to have fast degradation rates if the rate was among the fastest 20% in both organisms, and vice versa for slow degradation rates.

To investigate if proteins that had slow and fast degradation rates, respectively, displayed differences in abundance, we performed Wilcoxon-Mann-Whitney test between the two groups of the ion intensities from the light label.

To investigate if proteins that had slow and fast degradation rates, respectively, displayed differences in disordered structure, we performed Wilcoxon-Mann-Whitney test between the two groups using the % disorder per protein as calculated from the human structure using disorder2 software(Ward *et al*, 2004).

For KEN motif analysis, we calculated the number of KEN motifs in each protein sequence using an in house algorithm in Matlab (matworks.com) that searched for the canonical KENXXX[NDEQ] motif(Pfleger & Kirschner, 2000). A Fisher’s exact test between the two populations was used to calculate significance.

### Data analysis for identification of synthesis and degradation rate of the proteins in macromolecular sub-complexes

Proteins were identified and quantified using MaxQuant with similar settings as above except label free quantitation was enabled. Chromatograms of the individual proteins were constructed using the label free quantitation value of the light label in each fraction, and by smoothing these curves using a moving average of three consecutive fractions in Matlab. The relative synthesis and degradation rates of the proteins in the individual fractions were calculated from the normalized values of H/L and M/L which was log_2_ and –log_2_ transformed, respectively.

### Identifying the correlation between mRNA and protein expression change

The mRNA dataset derived from (Suzuki *et al*, 2009)was filtered for a detection value larger than 0.95. The counts for 6, 12, 24 and 48 h differentiation were divided by the counts at 0 h before the ratios were log_2_ transformed. Changing mRNA was defined similarly to the proteins (see above) using Perseus. Gene-name identifiers were used to pair mRNA and protein expression values.

The Spearman correlation was calculated using Perseus between mean for the mRNA and proteins expression values for the gene having two out of three expression values in both datasets. Changing genes was defined as changing expression both on the mRNA and protein level using the above criteria.

The Spearman correlation between synthesis rate and mRNA and protein expression was calculated using the data from the three biological replicates of each of the parameters resulting in nine correlation values each, from which we could draw boxplots.

2D enrichment analysis (P<0.05) of the biological processes (Uniprot Keywords) that were enriched in any dimension of protein or mRNA expression change after 48 h THP-1 differentiation were generated using Perseus similar to (Cox & Mann, 2012) with a Benjemini-Hochberg FDR for truncation.

### Partial least square regression analysis

The partial least square regression (PLS) analysis was performed in Matlab using the *plsregress* function. We calculated PLS regression for genes that had transcriptome expression data after 48 h differentiation, proteome expression data after 48 h differentiation and synthesis and degradation rate data in two out of three biological replicates using the mean of the values; the increasing and decreasing proteins were defined as in the expression experiment as above. The P-values in supplementary table 6 was calculated by the pls-package in R.

### Determining the effects of perturbation on degradation and synthesis rates

The correlations between synthesis and degradation rates in differentiating versus proliferating cells were calculated using the data from the three biological replicates of each of the parameters resulting in nine correlation values each, from which we could draw boxplots.

Biological processes (Uniprot Keywords) that were enriched (P<0.05) in any data dimension of synthesis or degradation rates between differentiating and proliferating cells were generated using Perseus and Benjemini-Hochberg FDR for truncation.

To produce the heatmap for the differentially regulated proteins and proteins with differentially regulated synthesis and degradation rates, we performed enrichment analysis of the biological processes (Uniprot keywords) using GPROX(Rigbolt *et al*, 2011) against the increasing and decreasing protein’s expression, synthesis and degradation rates, using Benjamini and Hochberg correction with a minimum of 5 proteins per category (P<0.05).

# References

Baek D, Villén J, Shin C, Camargo FD, Gygi SP & Bartel DP (2008) The impact of microRNAs on protein output. *Nature* **455:** 64–71

Cheadle C, Vawter MP, Freed WJ & Becker KG (2010) Analysis of Microarray Data Using Z Score Transformation. *The Journal of Molecular Diagnostics* **5:** 73–81

Cox J & Mann M (2008) MaxQuant enables high peptide identification rates, individualized p.p.b.-range mass accuracies and proteome-wide protein quantification. *Nature Biotechnology* **26:** 1367–1372

Cox J & Mann M (2012) 1D and 2D annotation enrichment: a statistical method integrating quantitative proteomics with complementary high-throughput data. *BMC Bioinformatics* **13:** S12

Gray KA, Daugherty LC, Gordon SM, Seal RL, Wright MW & Bruford EA (2012) Genenames.org: the HGNC resources in 2013. *Nucleic Acids Research* **41:** D545–D552

Kristensen AR, Gsponer J & Foster LJ (2012) A high-throughput approach for measuring temporal changes in the interactome. *Nature Methods* **9:** 907–909

Pfleger CM & Kirschner MW (2000) The KEN box: an APC recognition signal distinct from the D box targeted by Cdh1. *Genes & Development* **14:** 655–665

Rappsilber J, Mann M & Ishihama Y (2007) Protocol for micro-purification, enrichment, pre-fractionation and storage of peptides for proteomics using StageTips. *Nature Protocols* **2:** 1896–1906

Rigbolt KTG, Vanselow JT & Blagoev B (2011) GProX, a user-friendly platform for bioinformatics analysis and visualization of quantitative proteomics data. *Molecular & Cellular Proteomics* **10:** O110.007450

Rogers LD & Foster LJ (2007) The dynamic phagosomal proteome and the contribution of the endoplasmic reticulum. *Proc. Natl. Acad. Sci. U.S.A.* **104:** 18520–18525

Ruepp A, Waegele B, Lechner M, Brauner B, Dunger-Kaltenbach I, Fobo G, Frishman G, Montrone C & Mewes H-W (2010) CORUM: the comprehensive resource of mammalian protein complexes--2009. *Nucleic Acids Research* **38:** D497–501

Selbach M, Schwanhäusser B, Thierfelder N, Fang Z, Khanin R & Rajewsky N (2008) Widespread changes in protein synthesis induced by microRNAs. *Nature* **455:** 58–63

Suzuki H, Forrest ARR, van Nimwegen E, Daub CO, Balwierz PJ, Irvine KM, Lassmann T, Ravasi T, Hasegawa Y, de Hoon MJL, Katayama S, Schroder K, Carninci P, Tomaru Y, Kanamori-Katayama M, Kubosaki A, Akalin A, Ando Y, Arner E, Asada M, et al (2009) The transcriptional network that controls growth arrest and differentiation in a human myeloid leukemia cell line. *Nat. Genet.* **41:** 553–562

Ward JJ, Sodhi JS, McGuffin LJ, Buxton BF & Jones DT (2004) Prediction and Functional Analysis of Native Disorder in Proteins from the Three Kingdoms of Life. *Journal of Molecular Biology* **337:** 635–645
